# A Perspective on Microneedle-Based Drug Delivery and Diagnostics in Paediatrics

**DOI:** 10.3390/jpm9040049

**Published:** 2019-11-15

**Authors:** Liliana R Pires, KB Vinayakumar, Maria Turos, Verónica Miguel, João Gaspar

**Affiliations:** 1 International Iberian Nanotechnology Laboratory, 4715-330 Braga, Portugal; liliana.pires@inl.int (L.R.P.); joao.gaspar@inl.int (J.G.); 2 University of Oviedo, 33006 Asturias, Spain; mariaturos95@gmail.com; 3 Department of Cell Biology and Immunology, Centro de Biología Molecular “Severo Ochoa”, 28049 Madrid, Spain; vmiguh00@gmail.com

**Keywords:** microneedles, medical devices, paediatrics

## Abstract

Microneedles (MNs) have been extensively explored in the literature as a means to deliver drugs in the skin, surpassing the *stratum corneum* permeability barrier. MNs are potentially easy to produce and may allow the self-administration of drugs without causing pain or bleeding. More recently, MNs have been investigated to collect/assess the interstitial fluid in order to monitor or detect specific biomarkers. The integration of these two concepts in closed-loop devices holds the promise of automated and minimally invasive disease detection/monitoring and therapy. These assure low invasiveness and, importantly, open a window of opportunity for the application of population-specific and personalised therapies.

Microneedles (MNs) are micrometre-scale structures with sharp tips that can perforate the upper layers of the skin, overcoming the *stratum corneum* barrier. MNs have been primarily explored in the literature as a means to deliver drugs through the skin layers [1,2], to detect and monitor specific molecules in the interstitial fluid [3,4] and to monitor cells in vitro [5]. Table 1 shows a comparison of different drug delivery techniques, including MN-based drug delivery. MNs are potentially easy to produce and allow self-administration and high patient compliance, as they cause no pain or bleeding [1,2,4,5]. Along with developments in MN research, the field of transdermal patches has grown. Although the transdermal administration of drugs has been considered very attractive and convenient, it has been limited by skin permeability to molecules with very specific characteristics, namely, those of small molecular weight and balanced hydrophobicity. With the application of MN arrays, the transdermal route becomes accessible to many other molecules. Some such examples are already in clinical trials [6]. The administration of vaccines, namely, influenza [7] and polio [8], already have published results from clinical tests. Other molecules such as zolmitriptan, a selective serotonin receptor agonist used for the treatment of migraine, and abaloparatide, a parathyroid hormone-related protein analog used to treat osteoporosis, are currently in phase III clinical trials (see [6]). 

Although its primary target is the skin, MNs are also being investigated as a mean to overcome other biological barriers, releasing drugs in the eye [9], the oral mucosa [10], the vaginal mucosa [11], vascular tissue [12] and so forth. Alternatively, MN patches have evolved to collect interstitial fluid painlessly, providing monitoring or detection of specific biomarkers [13]. This field is growing and holds the promise of allowing minimally invasive disease monitoring, ultimately integrating closed-loop devices in which detection and therapy are achieved in a minimally invasive way. Taking into consideration its specificities and irrefutable advantages, the field of MN research is currently evolving towards personalised medicine, developing treatments for specific populations, such as the elderly and children.

The use of MN patches brings particular advantages that may have a huge impact on the field of paediatrics. These include painless and safe drug administration; minimising the risk of bleeding, infections and injuries; and favouring therapy acceptance among children and also parents [14]. Moreover, MNs have been considered particularly promising for the administration of vaccines, taking advantage of the role of the skin in the immune system. The skin is not only a physical barrier but also a complex and active immune site, highly rich in antigen-presenting cells, including macrophages, Langerhans cells and dendritic cells. These cells play a significant role in adaptive immune responses, converting the skin into a favourable place for immunisation [15]. Therefore, theoretically, the use of MN patches provides important advantages compared with intramuscular or subcutaneous vaccine administration. Different studies using animal models demonstrated increased immunogenicity of vaccines when administered via MN patches compared with conventional administration [16,17]. However, this has not yet been confirmed in clinical trials [7].

Most vaccines, such as polio, diphtheria, tetanus or pertussis, are administered in the first year after birth and during childhood. Vaccination using a conventional needle system often poses challenges for both parents and medical staff due to needle phobia and pain. The dosage and time window vary according to the country vaccination program, but MNs can have a major positive impact on childhood vaccination (Figure 1B), as already demonstrated by the positive perception from parents, children and medical staff [18].

The different characteristics of child/infant skin compared with adult skin may represent an extra challenge for researchers to implement the MN-based system. The needle specifications need to be tailored considering the skin cross section and mechanical properties of the paediatric skin [19]. Children’s skin is thinner (Figure 1A) compared with adult skin and may require new and deeper studies on the pharmacodynamics of transdermal drug dissolution and diffusion. However, some studies have reported no differences in skin thickness depending on age, gender or body mass index in children below 5 years old; and although they found variable thicknesses in different areas of the body, these were found to have no clinical relevance. Even so, Duarah et al. [19] pointed to the fact that the skin represents 3% of the body weight in adults and is 13% in a preterm infant. Thus, the area of application of a transdermal patch may have relevant consequences regarding safety among these groups. In terms of mechanical stability, it is considered that children’s top layer of skin (Figure 1A) can be disrupted with a needle height of around 300 μm and a diameter of less than 100 μm [19]. In terms of dosage, a lower dosage requirement in paediatrics is expected, which may make it easier to achieve therapeutic doses through MN patches. These parameters have to be addressed by designing suitable microneedle architectures to target children of different age groups (infants, children and adolescents).

The use of MN patches holds the promise of finding applications other than vaccine therapeutics for children, namely, in high-incidence-rate diseases and diseases that can benefit from administration through the skin, such as immune-related diseases. Psoriasis is a chronic autoimmune disease in which the life cycle of skin cells is accelerated, producing “extra” skin. This forms scales and red patches that are itchy and sometimes painful. In children, the incidence rate is increasing, reaching 2% in some populations [20]. One of the main challenges posed by chronic diseases is ensuring patient adherence to therapy. In diseases that start in early childhood, this becomes a considerable issue. Administration of these therapies through MNs is expected to increase patient compliance [21] and, consequently, provide significant improvements in treatment effectiveness. One such example is growth hormone deficiency, a genetic disease that requires life-long growth hormone administration. The administration of the hormone via MN patches is considered very promising, providing similar bioavailability compared to subcutaneous injection and a patient-friendly alternative [22]. Paediatric asthma is the most common serious chronic disease in infants and children. This noncommunicable disease affects 11.1%–11.6% of children worldwide [23]. It is an inflammatory disease of the airways in the lungs, marked by attacks of spasm in the bronchi, causing difficulty in breathing. MN patches can improve the therapeutics of asthma [24]. It is also expected that microneedle devices can provide a new solution for the treatment of diabetes. Diabetes also has a high incidence rate in childhood. In the United States, it is estimated that about 193,000 people have diagnosed diabetes under age 20, and more than 20% have type II diabetes [25]. MN-based devices not only have the potential to provide the administration of insulin to diabetic patients [26] but also to monitor the disease by assessing glucose [27,28], hopefully in a fully integrated and automatic system [29,30], as represented in Figure 2B.

As previously mentioned, in light of applications among the paediatric population, MN patches should highly control the release of specific drugs. Different MN designs and materials have been applied to different applications, providing different drug release kinetics. Administration of fast-acting drugs can be achieved by the use of coated MNs, in which the drug molecule is adsorbed on the MN surface. Upon insertion, the coated drug on the outer wall will diffuse through the skin layers. Some studies have applied polymers along with the drug molecule to be delivered in order to protect it during skin penetration [31,32]. Alternative methods to circumvent the shear force effect in coated microneedles have been previously proposed using pocketed MNs, grooved MNs and cup-shaped MNs [31,33,34]. Similarly, in the field of polymeric MNs, fast-dissolving polymers, such as polyvinylpyrrolidone and sugars, have been used along with drugs for rapid dissolution/diffusion [35,36]. Recently, polymeric MNs were applied in the area of controlled long-term release applications. This has been explored using polymers with low water solubility or low degradation rates [37]. Precise control over the drug release can be obtained using triggered drug release methods. An example was described by Lee et al., who applied an electroresistive heater connected to MNs containing a specific drug and coated with thermosensitive polymers. In this study, detection of high glucose levels triggered the activation of the heater. Consequently, the polymer dissolved and the antidiabetic drug was released [29]. Alternatively, pH-triggered release has also been explored [16]. Nanotopography on MN patches has been used as a means of increasing transdermal delivery of high-molecular-weight drugs. The nanotopography affects the delivery via an integrin-dependent mechanism, altering how cells interact with the MNs and increasing paracellular permeability [38].

The development of effective MNs for paediatric applications will have a broad range of uses in the field of disease monitoring, in which MNs can be applied to collect interstitial fluid painlessly. The sampled interstitial fluid can subsequently be analysed by lab-on-a-chip devices or wearable diagnostics to measure different analytes (Figure 1C). Situations such as type 1 diabetes and hepatitis B require the frequent collection of blood/interstitial fluid samples for the quantification of glucose or viral antigens, respectively. The application of wearable devices that can detect and monitor these molecules can be enabled by the use of MNs. An elegant approach explores the use of polymeric hydrogel-forming MNs that soak the interstitial fluid (Figure 2A) [39]. The fluid is subsequently collected for the detection of glucose and cholesterol. Comparable quantification of these molecules has been demonstrated in the interstitial fluid and blood [40].

Looking forward, there is a potential market for MN-based closed-loop drug delivery systems. As shown in Figure 2B, we envisage integrated closed-loop systems in which one MN patch is used to draw the interstitial fluid and another to deliver the required drug in a minimally invasive manner. The drawn interstitial fluid through the MN patch is driven towards a biochemical sensor to analyse a specific biomarker. The development of biochemical sensors has been a highly acclaimed research area over the last two decades and a variety of biosensing strategies have been reported to detect biomarkers from the blood, interstitial fluid, sweat, tears and so forth. [44,45]. For continuous and controlled drug delivery, along with MNs, micropumps have gained particular attention. In our previous work, we demonstrated the integration of a peristaltic micropump with a hollow MN array to deliver insulin [46,47]. Although promising, these systems still need further improvements, namely, in the miniaturisation of the drug delivery pump [48,49]. These pumps need to ensure two specific characteristics: (1) the pump should work with a constant flow rate against the blood pressure and during power failures, and (2) the pump should provide a closed path between the drug reservoir and the bloodstream. In a closed-loop approach, continuous monitoring of biochemical markers and continuous drug delivery will be achieved by integrating hollow MN arrays with biochemical sensors and miniaturised micropumps [21]. Although solid microneedles (polymeric/soluble/coated) can accommodate clinically relevant amounts of a specific drug, for long-term applications, devices based on hollow MNs and micropumping systems will be required.

The field of microneedles is now crossing borders between disciplines towards fully integrated medical devices. This is a promising way towards creating new solutions in healthcare, which we envisage to have a huge impact on procedures for assessing and treating paediatric patients. 

## Figures and Tables

**Figure 1:**
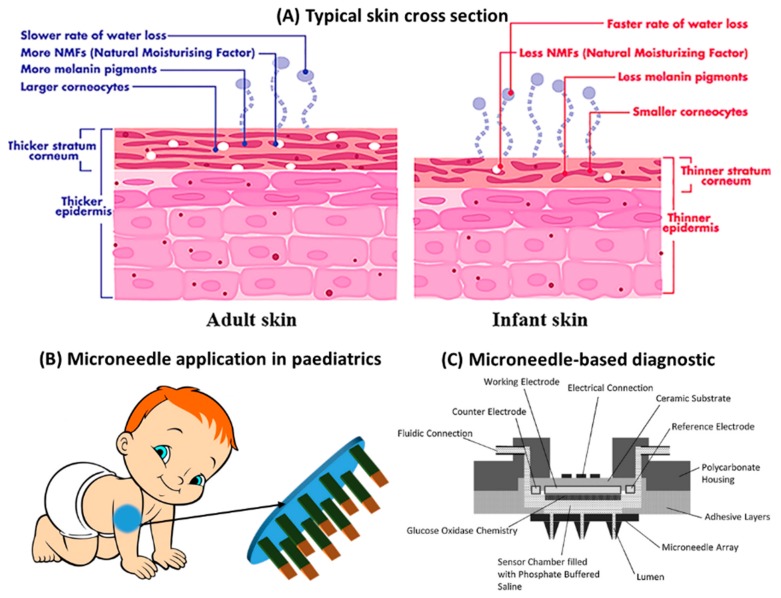
(**A**) Typical cross section of adult skin and infant skin [19]. (**B**) Proposal for the potential microneedle application in paediatrics. (**C**) Microneedle-based diagnostics to monitor the required analyte; microneedles are used to sample the interstitial fluid in a painless manner [13].

**Figure 2:**
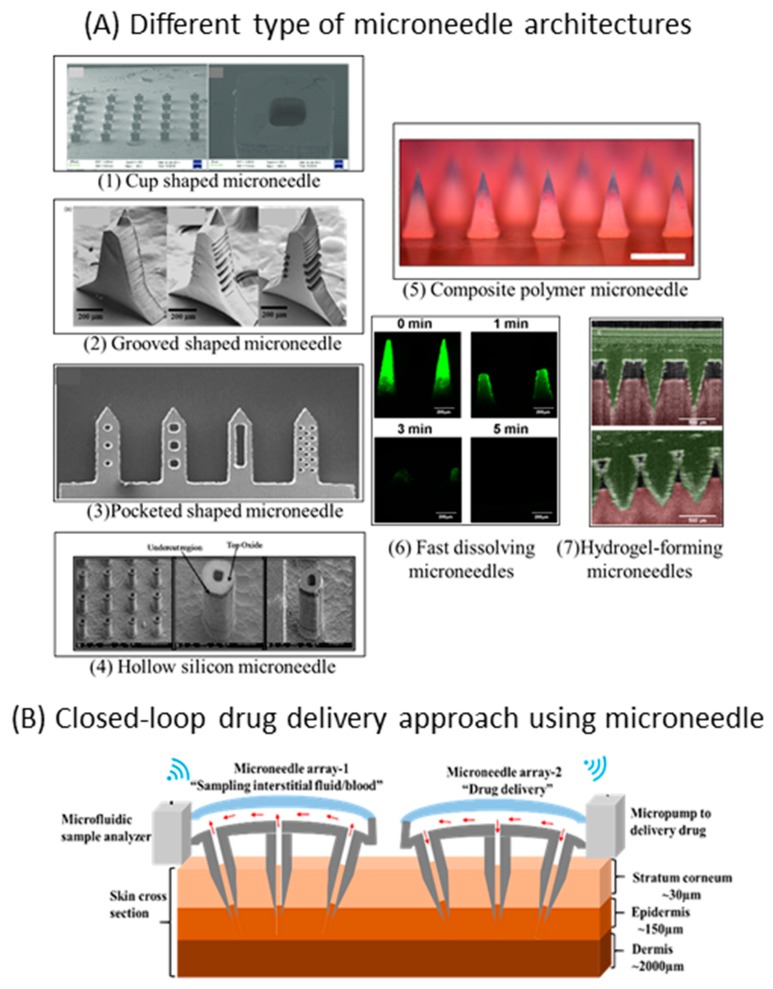
(**A**) Different microneedle architectures used to deliver drugs and to sample interstitial fluid. Scanning electron microscopy micrographs of (**1**) cup-shaped MNs [33], (**2**) groove-shaped MNs [34], (**3**) pocketed MNs [31] and (**4**) hollow MNs [41]. Fluorescence microscopy images of (**5**) polymer MNs [42] and (**6**) fast-dissolving polymeric MNs [43]. (**7**) Optical coherence tomography images of hydrogel MN following insertion into excised neonatal porcine skin [18]. (**B**) Schematic shows the microneedle-based approach towards continuous monitoring and drug delivery as a potential closed-loop device.

**Table 1:** Comparison table of different drug delivery technologies.

	Advantages	Disadvantages
**Hypodermic needles**	-Direct access to the circulatory system (intravenous)	-Painful -Tissue damage -Nonautonomous administration
**Needless drug delivery**	-Rapid absorption (sublingual) -Economical, high dose possible (oral)	-Small dose limit -Jet may be painful
**Inhalation**	-Bypasses liver -Large surface of absorption	-Difficult to regulate the exact amount of dosage -Difficult to verify in infants
**Microneedles (MNs)**	-Pain-free administration -**Easy to use** -Continuous and controlled release -**Safer handling** -**Self-administration**	-Local inflammation -Skin irritation
